# De Novo Chromosome-Level Genome Assembly of ‘Qing Zhou Mi’ Landrace Peach and Analysis of Late Maturity and Fruit Weight Traits in Peach

**DOI:** 10.3390/plants15071113

**Published:** 2026-04-03

**Authors:** Miao Li, Qingtao Gong, Guixiang Li, Jing Gao, Anning Zhang

**Affiliations:** 1 National Key Laboratory of Efficient Utilization of Nutrient Resources, Shandong Academy of Agricultural Sciences, Jinan 250000, China; limiao6543@163.com (M.L.); gongzheng.1984@163.com (Q.G.); liguixiang@shandong.cn (G.L.); 2 Shandong Institute of Pomology, Longtai Road No.66, Taian 271000, China; 3 Weifang Academy of Agricultural Science, Weifang 261000, China; jmgaojing@163.com

**Keywords:** peach landrace, genome assembly, resistance, ethylene, GWAS

## Abstract

‘Qing Zhou Mi’ (QZM) is a typical representative landrace of the late-ripening, high-resistance, and small-fruited peaches found in northern China. However, its genetic information has not been systematically analyzed. In this study, we sequenced and de novo assembled the QZM genome. The chromosome-level genome was 252.39 Mb in size, with a contig N50 of 24.35 Mb. Comparative genomic analysis found a total of 9.24 Mb unique fragments and 418 genes in the QZM genome, most of which were associated with resistance. Compared with the genomes of some early maturing peach accessions, the differentiation ability of the ACC oxidase and ethylene receptor gene families related to ethylene synthesis and transport in QZM was significantly weakened. In the genome-wide association study, we identified *PpNAC1* as a major gene regulating the late-ripening trait of QZM. In addition, we discovered a novel locus associated with fruit weight and focused on a candidate gene in its regulation, *PpLOB33*. The findings of this study can serve as a foundation for further research on the genetic basis underlying the core traits of QZM, providing precise targets for molecular breeding.

## 1. Introduction

The study of plant genomics improves our understanding of the genetic basis of plant diversity. Since the publication of the *Arabidopsis* genome in 2000 [1], the development of plant genomics over the past 20 years can be divided into two stages. During the first stage, due to the limitations of sequencing technology, researchers achieved the genome sequencing and assembly of model terrestrial plant species or cash crops with small diploid genomes [2,3]. The study of plant genomics has now entered the second stage with the further development of sequencing technology, and the amount of research on plant genome sequencing has rapidly increased, with sequenced samples expanded to include plants with special phenotypes, medicinal plants, and endangered plants [4,5,6].

The first assembled peach genome was the western rootstock ‘Lovell’ [7]. In recent years, with the rapid development of third-generation sequencing technology, the genomes of selected peach varieties have been assembled for the analysis of important agronomic traits. For example, Yu et al. [8] generated the genome of ‘Longhuashuimi’ (LHSM), a representative of the Chinese cling peaches, and identified two key genes, *PpALMT1* and *PpERDL16*, that were associated with fruit flavor. In another study, the genome of a flat-fruit peach cultivar, ‘Rui You Pan 1’ (RYP1), was assembled, and a 1.67 Mb heterozygous inversion was found to influence the flat fruit shape [9]. The genome of Chinese cling peach, an important founder cultivar for peach breeding programs, has also been generated, and 25 quantitative trait loci related to seven volatile compounds have been identified [10]. The draft genome of the flat peach cultivar ‘124 Pan’ was assembled in another study, and the expansion of genes involved in terpene biosynthesis was analyzed [11]. Other work assembled a high-quality genome of ‘Zhongyoutao 14’ (CN14), and *PpTIP2* was found to be associated with temperature sensitivity in this semi-dwarf cultivar [12]. The de novo genome assembly of four wild relatives of peach has also been performed, providing detailed basic data to support the study of peach evolution and dissect the genetic mechanism of resistance traits [13]. In addition, the genome of an ancient representative peach landrace, ‘Feichenghongli’, has been released, and key genes controlling late fluorescence and narrow leaf traits in this landrace have been identified [14].

Ethylene is a plant hormone that plays a core regulatory role in the fruit ripening process, influencing the entire cycle of ripening initiation, quality formation, and senescence [15,16]. The ethylene biosynthesis process in plants begins with methionine. First, methionine is catalyzed by S-adenosylmethionine synthetase (SAMS) to form S-adenosylmethionine (SAM). Then, in the rate-limiting step, SAM is catalyzed by ACC synthase (ACS) to produce 1-aminocyclopropane-1-carboxylic acid (ACC), which is the direct precursor of ethylene. Finally, ACC is converted into ethylene under the action of ACC oxidase (ACO). ACS is a rate-limiting enzyme in the ethylene biosynthesis pathway that directly determines the rate of ethylene synthesis. In tomato, increasing the expression of *ACS* genes such as *ACS1A*, *ACS2*, and *ACS4* promotes ethylene production, thereby accelerating fruit ripening [17]. *ACOs* also play an important role in the ethylene synthesis pathway. The expression level of *PpACO1* in peaches increases significantly during fruit ripening, which promotes ethylene synthesis and thereby drives fruit ripening [18].

Fruit softening is a crucial process that affects the post-harvest quality and storage life of fruits, and plant polygalacturonase (PG) genes play a key role in this process [19]. The main functions of PG genes include participating in pectin degradation and regulating cell wall metabolism. In strawberries, the expression level of the *FxaC_21g15770* gene increases significantly during the fruit ripening and softening stages, and silencing this gene can significantly enhance the firmness of strawberry fruits [20]. In strawberries, after silencing the *FaPG1* gene, the expression of many genes encoding cell wall-modifying enzymes is downregulated, which indicates that PG genes hold an important regulatory position in the cell wall metabolic network [21].

As the origin of peaches and a major center of peach domestication, China possesses extremely rich peach germplasm resources and has established long-term and systematic peach breeding programs. National peach germplasm repositories have been constructed to conserve thousands of accessions, including wild relatives, landraces, and improved cultivars [8,10]. Over recent decades, China’s breeding objectives have gradually shifted toward peaches with a high sugar content, low acidity, attractive appearance, extended maturity period, good disease resistance, high abiotic stress tolerance, and good suitability for mechanical harvesting and protected cultivation. Meanwhile, conventional hybridization has been combined with advanced molecular breeding technologies such as genomic selection, pan-genome analysis, and gene mapping [11,12,14]. These efforts have successfully bred hundreds of new peach cultivars with excellent agronomic traits, supporting the sustainable development of the peach industry in China.

The landrace peach ‘Qing Zhou Mi’ (QZM) originated in Qingzhou City, Shandong Province, China, and has been cultivated for more than 400 years. The fruits feature a small fruit shape, a pleasantly sweet flavor, extremely late maturity, and a long storage period. In addition, QZM has excellent resistance and is widely utilized as a rootstock in the peach industry of Shandong Province. With its long cultivation history and seed propagation, some individuals with phenotypic variations have been generated in the progeny population of QZM. In this study, we sequenced and de novo assembled the genome of QZM (252.39 Mb). We compared the assembled genome with previously released peach genomes to identify the genome specificity of QZM. In addition, we performed a genome-wide association study (GWAS) of 145 peach accessions (including 29 QZM variant accessions) to explore the key genes involved in fruit weight and fruit ripening period. This study aims to address the lack of systematic analysis of the genetic information of QZM and fill the gap in research on the genetic background of this distinctive local peach variety. Additionally, it is designed to provide theoretical support for exploring the genetic mechanisms underlying core agronomic traits in peach, to identify precise targets for peach molecular breeding, and to promote the directional breeding of peach varieties with elite traits, such as late maturity and high resistance.

## 2. Results

### 2.1. QZM Genome Sequencing, Assembly, and Assessment

**Figure 1:**
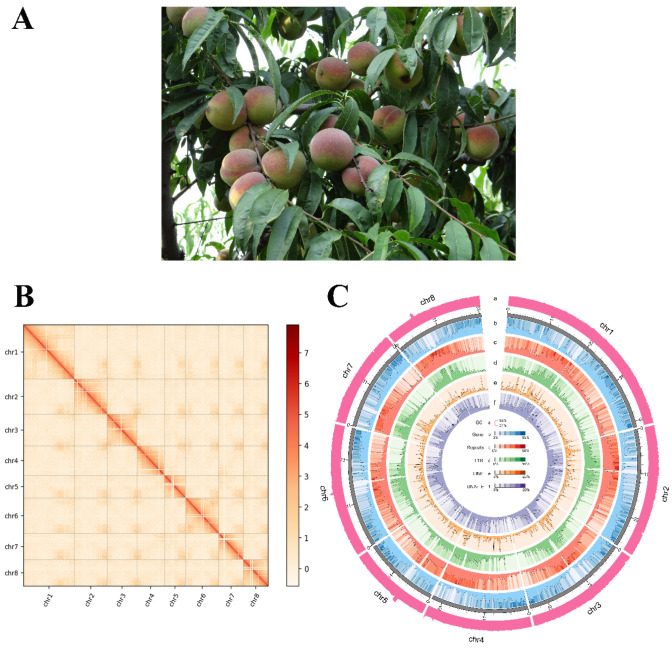
Characterization of the ‘Qing Zhou Mi’ (QZM) landrace peach genome. (**A**) Mature fruit phenotype of QZM. (**B**) Hi-C interactions among eight chromosomes (100 kb resolution). Strong interactions are indicated in dark red, and weak interactions are indicated in white. (**C**) Features across the QZM genome. Tracks (outer to inner circles) indicate (a) GC content, (b) gene density, (c) repeats, (d) LTRs, (e) lines, and (f) transposable elements.

The genome of QZM was de novo assembled using 118× coverage of 29.84 Gb of HiFi reads, 100× coverage of 25.28 Gb of Illumina short reads, and 106× coverage of 27.18 Gb of Hi-C data. Based on k-mer analysis (k = 17), the estimated genome of QZM was 255 Mb, with a heterozygosity of 0.49%. Eight chromosomes and 194 scaffolds were constructed using the original 213 contigs (contig N50 = 24.35 Mb). The chromosome anchoring rate was 93.79%, and the assembled genome size reached up to 252.39 Mb, which accounted for 98.9% of the total assembled sequences (Figure 1).

The assembled QZM genome was assessed using three strategies. First, the second- and third-generation data were compared with the assembled genome, and the mapping rate and coverage reached up to 99.86% and 99.96%, respectively. Second, to evaluate the genome continuity, the LTR assembly index (LAI) was used, with the QZM genome assembly exhibiting a good LAI score of 14.55. Third, the high percentage of complete BUSCO genes (99.07%, 1599 of 1614) aligned to the QZM genome. Collectively, these findings confirm that the assembled QZM genome can serve as a good reference for further investigation of the molecular biology of peach.

### 2.2. Genome Annotation

Repeat sequences were identified using TRF (v4.09.1), RepeatMasker (v4.0.9), RepeatProteinMask (v4.0.9), RepeatModeler (v2.0.5), and LTR-FINDER (v1.07) software [22,23,24,25]. After the removal of redundant repeat sequences using the software, a total of 99.16 Mb repeat sequences (39.29%) in the QZM genome were detected (Tables S1 and S2). Gene prediction was performed using a strategy of homologous prediction from 10 libraries for related species. Finally, a total of 26,259 genes (98.31%) were annotated. Noncoding RNAs (ncRNAs) in the QZM genome were also annotated, yielding 130 miRNAs (0.006%), 571 tRNAs (0.017%), 7995 rRNAs (1.571%), and 472 snRNAs (0.022%).

### 2.3. Unique Fragments of the QZM Genome Compared with Four Different Peach Genomes

To identify unique fragments of the QZM genome, we conducted comparative analysis utilizing four additional re-sequenced peach genomes: 124Pan, CN14, Lovell, and *Prunus ferganensis*. The results yielded a total of 1939 fragments containing 9,247,663 bp (Table 1), as well as 418 genes. Among them, the proportion of the number of aligned fragments on the chromosome accounted for 81.03% of the total (1571/1939), and the proportion of their length accounted for 25.41% (2,350,054 bp/9,247,663 bp). The proportion of the number of unaligned fragments on the chromosome accounted for 18.97% (368/1939), and the proportion of their length accounted for 74.59% (6,897,629 bp/9,247,663 bp).

**Figure 2:**
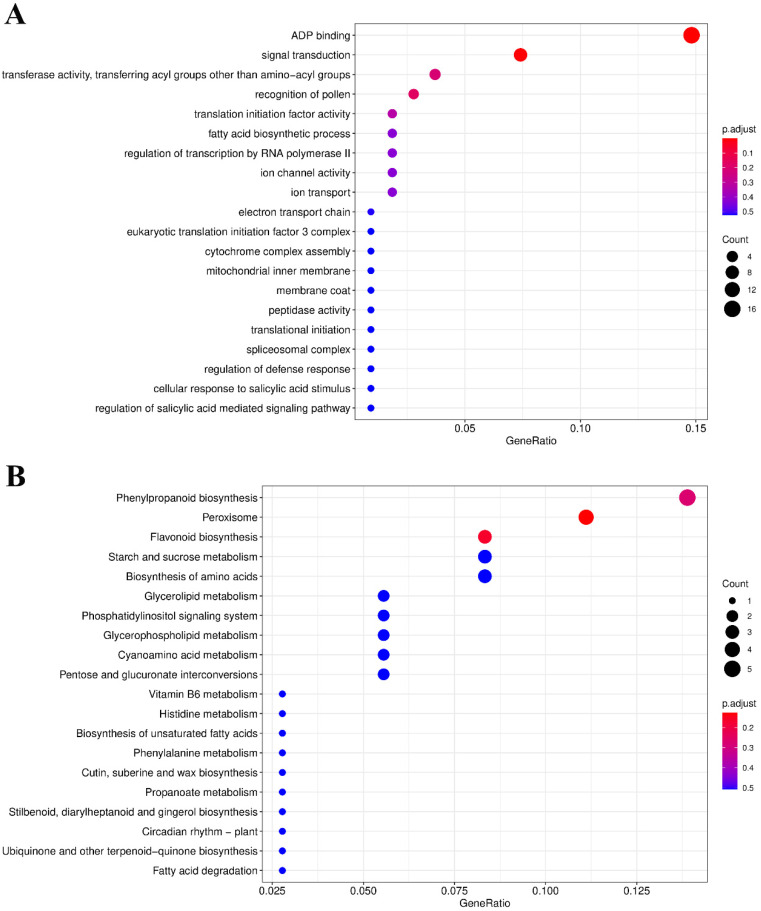
Top 20 Gene Ontology (GO) terms (**A**) and Kyoto Encyclopedia of Genes and Genomes (KEGG) terms (**B**) for genes in specific fragments.

To further investigate the functional characteristics of 418 genes in the unique fragments of the QZM genome, Gene Ontology (GO) functional enrichment analysis and Kyoto Encyclopedia of Genes and Genomes (KEGG) pathway enrichment analysis were performed. The top 20 terms in the GO analysis were mainly enriched in ADP binding, signal transduction, and recognition of pollen (Figure 2A). In the KEGG analysis, the top 20 terms were primarily enriched in peroxisome, flavonoid biosynthesis, and phenylpropanoid biosynthesis (Figure 2B).

### 2.4. Expansion and Contraction of Gene Families in the QZM Genome

**Figure 3:**
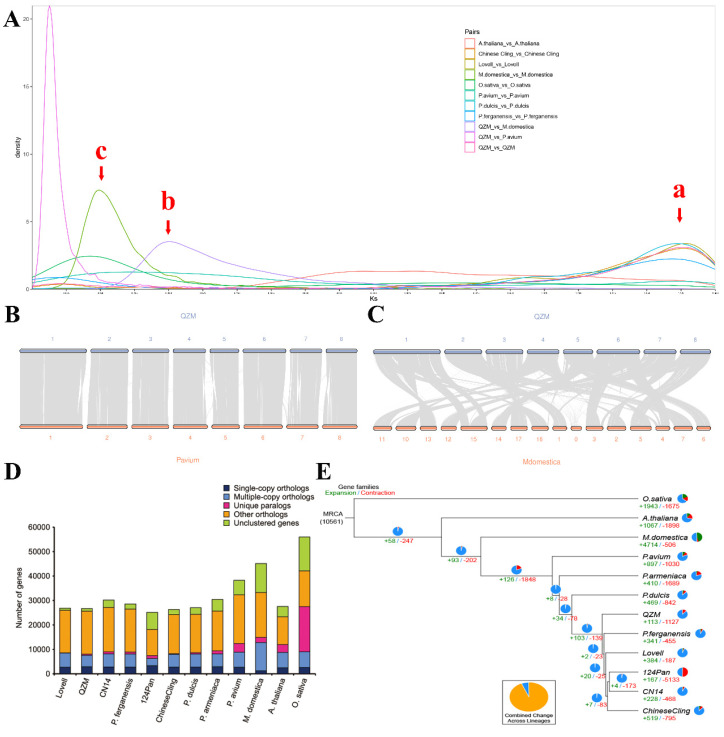
(**A**) Synonymous substitution rate (Ks) distribution map of 12 genomes (‘Lovell’, *Prunus ferganensis*, ‘Chinese cling’, ‘124Pan’, ‘CN14’, *Prunus dulcis*, *Prunus armeniaca*, *Prunus avium*, *Malus domestica*, *Arabidopsis thaliana*, and *Oryza sativa*). The peak in the plot produced by intraspecific combinations indicates the whole-genome duplication event of the species itself; the peak in the plot generated from interspecific combinations represents species divergence. a, the γ early duplication event; b, the divergence times of QZM vs. *M. domestica*; c, the divergence times of QZM vs. *P. avium*. (**B**) Collinearity analysis of ‘Qing Zhou Mi’ (QZM) landrace peach vs. *P. avium* based on coding sequences. The numbers in the figure represent the chromosome numbers. (**C**) Collinearity analysis of QZM vs. *M. domestica* based on coding sequences. The numbers in the figure represent the chromosome numbers. (**D**) Statistical results for homologous gene numbers of 12 species. (**E**) Phylogenetic tree and gene family expansion and contraction of 12 species. MRCA, most recent common ancestor. Green numbers indicate the number of expanded gene families, and red numbers indicate the number of contracted gene families during the evolution of the species.

To detect whole genome duplication (WGD) events in 12 genomes comprising six peach varieties (Lovell, *P. ferganensis*, Chinese cling, 124Pan, and CN14), four closely related crops (*Prunus dulcis*, *Prunus armeniaca*, *Prunus avium*, and *Malus domestica*), and the non-Rosaceae plants *A. thaliana* and *Oryza sativa*, the nucleotide diversity was examined using the synonymous substitution rate (Ks) method. A shared peak (Ks = 1.42) was detected in 11 genomes, excluding *A. thaliana*, which indicated that most species underwent the γ early duplication event (Figure 3A; arrow a). In addition, two distant peaks were detected, representing the divergence times of QZM vs. *M. domestica* (Ks = 0.30; arrow b) and QZM vs. *P. avium* (Ks = 0.15; arrow c). The divergence time of QZM vs. *M. domestica* was earlier than that of QZM vs. *P. avium*, which suggested that QZM shared a closer genetic relationship with *P. avium* than *M. domestica*. To further investigate the genome evolution, we examined the collinear relationships between QZM and the closely related species *M. domestica* and *P. avium* (Figure 3B–C). The results revealed a one-to-one syntenic block pairwise relationship between QZM and *P. avium*. However, the collinear relationships between QZM and *M. domestica* displayed numerous rearrangements and translocations of chromosome fragments. The results also indicated that QZM had a closer genetic relationship with *P. avium* than *M. domestica*.

To analyze the evolution of QZM at the genome level, 12 genomes encompassing six peach varieties (Lovell, *P. ferganensis*, Chinese cling, 124Pan, and CN14), four closely related crops (*P. dulcis*, *P. armeniaca*, *P. avium*, and *M. domestica*), and the non-Rosaceae plants *A. thaliana* and *O. sativa* (serving as the outgroup) were collected. The 12 genomes contained 4250 common families and 688 single-copy genes, with the number of unique families per genome ranging from 12 to 2494 (Figure 3D). A phylogenetic tree of the 12 species was constructed based on the 688 single-copy genes (Figure 3E). As expected, the six peach varieties were clustered into a single group in the resulting tree. Within this group, the distribution of QZM revealed a distant genetic relationship between QZM and the other five peach varieties. The four closely related crops were distributed on the exterior of the peach group.

To explore the evolutionary details of the QZM gene families, the expansion and contraction of gene families across 12 genomes were analyzed (Figure 3E). Among the six peach genomes, the QZM genome contained the fewest expanded families (113) and the second-largest number of contracted gene families (1127). We selected 51 gene families with significant expansion (286 genes) and 63 gene families with significant contraction (479 genes) (*p* < 0.05) for KEGG enrichment analysis (Figures S1 and S2). Enrichment was mainly detected in the plant–pathogen interaction, ABC transporters, and flavonoid biosynthesis pathways in the contracted gene families, while the oxidative phosphorylation, photosynthesis, and ribosome pathways were the predominant enriched pathways in the expanded gene families.

In addition, we screened for genes undergoing positive evolution under natural selection pressure (Ka/Ks > 1) throughout the QZM genome, and a total of 46 positively selected genes were identified (Table S3). Among these genes, chromosome 6 contained the largest number of positively selected genes (11 genes), suggesting that chromosome 6 underwent relatively strong natural selection pressure. Chromosomes 5 and 8 contained the fewest positively selected genes (two genes), implying that these two chromosomes underwent relatively weak natural selection pressure compared with the other chromosomes.

### 2.5. Key Members in the Ethylene Biosynthesis Pathway Across the QZM Genome and Other Peach Genomes

Ethylene plays a crucial regulatory role in fruit ripening. To investigate the ethylene biosynthesis pathway, *ACS* and *ACO* genes from the QZM genome and two other genomes of mid-maturing varieties, 124Pan and *P. ferganensis*, were identified (Table 2). The results showed that the three genomes contained the same number of *ACS* genes, but the number of *ACO* genes was fewer in QZM (29 genes) than in 124Pan (31 genes) and *P. ferganensis* (33 genes), which reflected a significant contraction in the QZM genome compared with the other two peach varieties. Ethylene receptor (*ETR*) family genes were also identified across the three genomes (Table 2), revealing that the QZM genome contained fewer *ETR* genes (three genes) than the 124Pan and *P. ferganensis* genomes, both of which contained four *ETR*s.

*PG* family genes are core genes that regulate fruit softening by degrading pectin. The fruits of QZM possess good storability. We further examined the differences between the *PG* family genes in the QZM genome and those of two peach varieties with poor storability, namely, CN14 and Lovell (Tables S4 and S5). The results showed that the number of *PG* genes was significantly fewer in the QZM genome (52 genes) than in the CN14 (68 genes) and *Lovell* (70 genes) genomes, which suggests that the relatively weak fruit softening found in QZM is correlated with the smaller number of *PGs* in its genome.

### 2.6. GWAS on the Agronomic Traits of QZM

**Figure 4:**
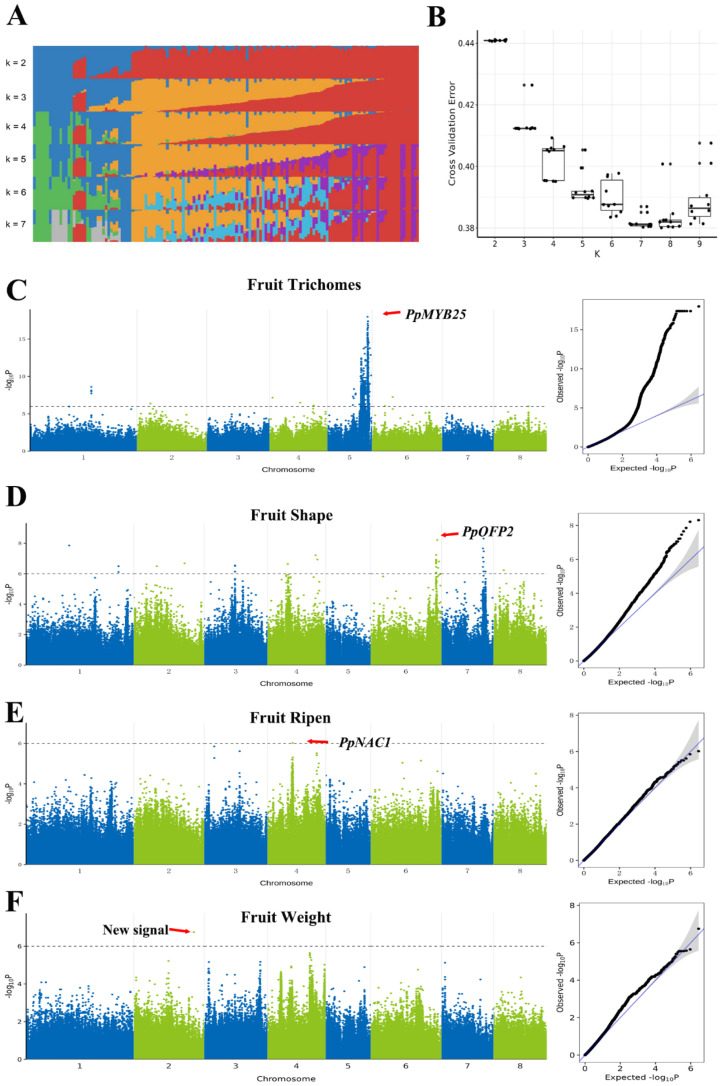
(**A**) Population structure of 145 peach accessions with different K values (ranging from 2 to 7). (**B**) Cross-validation errors of the re-sequenced population with different K values (ranging from 2 to 9). (**C**) Manhattan plot (**left**) and QQ plot (**right**) of the genome-wide association study (GWAS) for fruit trichomes and the corresponding peak signal on chromosome 5. (**D**) Manhattan plot (**left**) and QQ plot (**right**) of the GWAS for fruit shape and the corresponding peak signal on chromosome 6. (**E**) Manhattan plot (**left**) and QQ plot (**right**) of the GWAS for fruit ripening time and the corresponding peak signal on chromosome 4. (**F**) Manhattan plot (**left**) and QQ plot (**right**) of the GWAS for fruit weight and the corresponding peak signal on chromosome 2.

The QZM landrace peach is famous for its distinct agronomic traits, such as its small fruit size and extremely late ripening. To reveal the genetic basis of these traits, a GWAS population encompassing 145 peach accessions was constructed, which contained 29 landrace peaches from Shandong Province and 116 other peach varieties (Table S6). The K values of the population structure were analyzed, and cross-validation error analysis demonstrated that K = 7 was the optimal result (Figure 4A,B). This indicated that there was no significant family differentiation in the selected peach accessions, making them suitable for the subsequent GWAS analysis.

Using the QZM genome assembled in this study as the reference genome, 145 samples were re-sequenced and then subjected to alignment analysis with the QZM genome, yielding a total of 1,412,216 single-nucleotide polymorphisms (SNPs). After associating SNP markers with the traits of fruit hairs, fruit shape, fruit ripening, and fruit weight, peak signals were detected for all four traits. The peak signal associated with fruit trichomes was distributed on chromosome 5, and the reported key gene *PpMYB25* was identified near this peak signal (Figure 4C). The peak signal associated with fruit shape was detected on the end of chromosome 6, which contained the reported key gene *PpOFP2* (Figure 4D). The peak signal associated with fruit ripening was detected on the middle of chromosome 4, which featured the reported key gene *PpNAC1* (Figure 4E). Finally, a novel peak signal associated with fruit weight was detected on chromosome 2 (Figure 4F). This signal did not overlap with any previously reported genes, suggesting that this signal represented a novel locus regulating fruit weight.

## 3. Discussion

With the gradual maturity of sequencing technologies and the continuous reduction in sequencing costs, research has shifted from a focus on plant phenotypic differences to exploring genomic data linking gene functions, evolutionary history, environmental adaptations, and production applications [9,12]. Sequencing technology has become an indispensable technical support in the field of plant science, providing molecular-level solutions to address global issues such as food security, ecological crises, and resource shortages. Next-generation sequencing technology can be utilized to determine the genome size of a specific peach variety. The long evolutionary history of peach has given rise to three main groups of peaches, namely wild varieties, local varieties, and modern cultivated varieties [26,27]. In the present study, we collected genomic information for selected sequenced peach varieties and identified the evolutionary trends of the peach genome.

**Figure 5:**
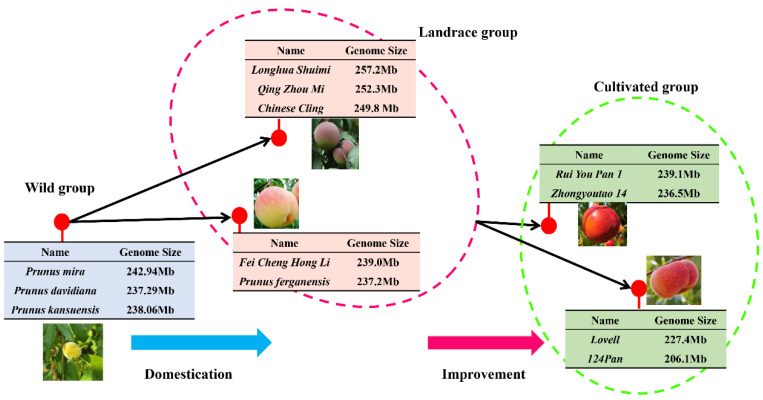
Genome size trends of selected sequenced peach accessions among the wild, landrace, and cultivated groups.

As shown in Figure 5, the genomes of re-sequenced wild varieties, including *Prunus davidiana* (237.2 Mb), *Prunus mira* (237.2 Mb), and *Prunus kansuensis* (238.0 Mb), were significantly smaller than those of landraces such as QZM (252.3 Mb), LHSM (252.3 Mb), and Chinese cling (249.8 Mb). This finding reflected the increase in genomic information during the early domestication of peach, which led to phenotypic changes such as enhanced fruit size and improved fruit quality [28,29]. However, not all landraces displayed a significant increase in genome size compared with wild varieties. The genome sizes of some landraces, such as Feichenghongli (239.0 Mb) and *P. ferganensis* (237.2 Mb), showed negligible expansion compared with those of wild varieties. Based on this finding, we infer that Feichenghongli and *P. ferganensis* retain a relatively large amount of genetic information from wild peaches and occupy a transitional position between the wild group and the landrace group. The genomes of landraces were significantly larger than those of modern cultivated varieties such as CN14 (236.5 Mb), 124Pan (206.1 Mb), and Lovell (227.4 Mb). This reflected the loss of genomic information during the subsequent improvement process, which led to changes in some phenotypes, such as the loss of fruit aroma and weakened stress resistance [26,27,30].

As a landrace variety, QZM has a long cultivation history of over 400 years. QZM not only possesses distinctive agronomic traits, such as small fruit size and good storage tolerance, but also exhibits uniqueness in its genome. In the present study, aligning the genome of QZM with four reported sequenced genomes enabled the identification of numerous unique fragments. Interestingly, only a quarter of these unique fragments could be aligned to eight chromosomes, while most of the remaining fragments were located in mitochondria and chloroplasts. We speculate that the genomic information in mitochondria and chloroplasts plays an indispensable role in regulating the development of the unique traits of QZM. Regarding the 418 genes on these unique fragments, we inferred the existence of several key genes based on gene annotation. For example, genes homologous to *Ppe_1G0015450* (annotation: putative disease resistance protein RGA1) have been shown to positively regulate resistance in wheat and cotton [31,32]. Therefore, we hypothesize that *Ppe_1G0015450* may contribute to the disease resistance of QZM. In addition, we detected important transcription factors, such as *Ppe_3G0008150* (annotation: transcription factor *MYB32*), for which the homologous gene *AtMYB32* was reported to positively regulate drought resistance in *Arabidopsis* [33]. Therefore, we speculate that *Ppe_3G0008150* may contribute to the drought resistance of QZM. Furthermore, KEGG enrichment analysis revealed that the functions of some genes were significantly enriched in the flavonoid and phenylalanine synthesis pathways, both of which positively regulated the secondary metabolic defense system, leading to enhanced plant resistance [29,34,35]. Overall, it can be seen that the excellent resistance of QZM may be closely related to its unique fragments.

Based on the ripening stage of fruit, the ethylene system in the late-ripening peach variety QZM was significantly less developed than that in the mid-ripening varieties 124Pan and *P. ferganensis*. Using comparative genomic methods, we analyzed the key enzymes in the ethylene biosynthesis pathway by comparing the QZM genome with the genomes of 124Pan and *P. ferganensis*. The results showed that the number of members in both the *ACO* and *ETR* families was notably lower in QZM than in 124Pan and *P. ferganensis*. We hypothesize that this underdeveloped expansion of ethylene-related gene families may be associated with the underdevelopment of the fruit’s ethylene system. Similarly, we found that QZM, with strong storage tolerance, contained significantly fewer *PG* family members than the storage-sensitive varieties CN14 and Lovell. This implies that the superior storage trait of QZM is associated with the smaller number of *PG* family members in its genome.

Utilizing GWAS analysis, we not only identified the key genes regulating the qualitative traits of fruit skin pubescence (*PpMYB12*) and fruit shape (*PpOFP2*) but also identified the major-effect gene *PpNAC1*, which controls quantitative traits. This demonstrated that the association analysis conducted in this study was scientific, accurate, and reasonable. Furthermore, we performed an association analysis of fruit weight and detected a significant peak signal, SNP_27296631_. No previously reported genes that regulate peach fruit weight were found in this region, indicating that this may represent a novel locus involved in the regulation of fruit weight. We searched for the significant signal SNP_27296631_ and identified one potential key gene associated with fruit development regulation, *Ppe_2G0023670*, which was annotated as LOB domain-containing protein 33, while the reported functions of its homologous genes include regulating cell cycle progression and influencing plant hormone signal transduction [36]. This provides a foundation for deciphering the regulatory mechanism underlying the small fruit trait of QZM.

## 4. Materials and Methods

### 4.1. Plant Materials and Sampling

Sequencing samples including roots, stems, leaves, flowers, and fruits were collected from the landrace peach QZM (tree with an age of over 30 years) in Yangjiawo village, Wangfen Town, Qingzhou City, Shandong Province, China (118.06° E, 36.04° N).

### 4.2. Genome Survey and Sequencing

Genomic DNA was isolated from QZM leaves using the cetyl trimethylammonium bromide method [37]. For short-read sequencing, fragmented DNA (−50 bp) generated *via* the S220 Focused-ultrasonicator (Covaris, Woburn, MA, USA) was adapter-ligated and sequenced on the Illumina NovaSeq X platform (Illumina Inc., San Diego, CA, USA) to produce 150 bp paired-end reads. Concurrently, a −20 kb SMRTbell library was constructed for PacBio sequencing. Following quality assessment (agarose gel electrophoresis and Thermo Fisher Scientific Qubit 4 fluorometry; Waltham, MA, USA), high-molecular-weight DNA was sheared to −15 kb using a g-TUBE (Covaris; Roswell, GA, USA) and size-selected with 0.45× AMPure beads (Beckman Coulter, Brea, CA, USA). Damaged ends were repaired enzymatically, and hairpin adapters were blunt-end ligated prior to sequencing on the PacBio Sequel II platform (P6-C4 chemistry; Menlo Park, CA, USA) at Shanghai OE Biotech Co., Ltd. (Shanghai, China).

### 4.3. Hi-C Library Construction

The Hi-C library was constructed following the standard protocol with minor modifications [38]. Briefly, nuclear DNA was cross-linked and digested to generate cohesive ends, which were then filled in with biotinylated nucleotides. The library was constructed by enriching for biotin-labeled products, followed by shearing to produce fragments of −350 bp. Sequencing was performed on an Illumina HiSeq X-Ten platform (Illumina, San Diego, CA, USA) to support the construction of chromosome-level pseudomolecules.

### 4.4. Genome Size Estimation

The genome survey was conducted using Jellyfish (v2.3.0) (parameters “-m 17 -C”) to analyze K-mer distribution [39]. Subsequently, GenomeScope (v2.0.1) was employed to estimate the genome size, heterozygosity rate, and repeat content of QZM [40].

### 4.5. Genome Assembly

High-fidelity HiFi reads were generated using the CCS sequencing mode (v4.2.0; https://github.com/PacificBiosciences/ccs (accessed on 15 October 2023)), which performed consensus correction on multiple subreads from the same SMRTbell molecule template. This yielded a total of 30.24 Gb of high-quality data. The QZM genome was subsequently assembled using Hifiasm (v0.14.2) [41].

### 4.6. Hi-C Scaffolding and Gap Filling

Sequencing yielded 189,278,390 raw reads, of which 184,752,308 high-quality clean reads were retained after filtering adapter and low-quality sequences using fastp v0.20.0 [42]. These clean reads were aligned to the contigs using BWA-mem v0.7.17 [43] with default parameters. The aligned reads were subsequently processed through Juicer v1.5.7 [44] and 3d-DNA v20180922 [38] for Hi-C analysis and scaffolding. The Hi-C contact matrix was visualized and manually corrected based on neighboring interactions using Juicebox v1.11.9 [44].

### 4.7. Repeat Annotation

We performed extensive de novo TE annotation using Annotator11 (EDTA v1.7.0) [45], which integrates LTRharvest from GenomeTools (v1.5.10) [46,47], LTR_FINDER (v1.0.7) [25], TIR-Learner (v2.4) [48], and HelitronScanner (v1.1) [49] to customize filtering scripts for each TE class (LTRs, TIRs, and Helitrons). Subsequently, LTR_retriever (v2.8.2) [50], RepeatModeller (v1.0.11) [51], and RepeatMasker (v4.0.9) [52] were employed to construct a comprehensive TE library by eliminating false positive LTR predictions and identifying previously undiscovered elements. Homology-based and structural annotations were conducted using RepeatMasker.

### 4.8. Gene Annotation

To annotate the genome, RNA-seq reads from five tissues (roots, stems, leaves, flowers, and fruits) were aligned using HISAT2 v2.10.2 [53] and reconstructed using StringTie v1.3.0. An equimolar RNA mixture from these tissues was used to generate a high-quality full-length transcript (Iso-seq) for enhanced annotation. Two rounds of MAKER v2.31.10 [54] prediction were conducted. The first round used BLAST v2.15.0 and Exonerate v2.4.0 to align assembled transcripts, homologous proteins, and ESTs to the genome, training Augustus [55] and GeneMark-ES [56] *via* BRAKER2 [57]. The second round integrated these data for final de novo gene prediction. BUSCO v3.1.0 [58] assessed annotation completeness. Functional annotation used Diamond (v2.1) [59] to assign gene functions based on the best matches in the NR, KOG, GO, Swiss-Prot, TrEMBL, eggnog, KEGG, InterPro, and Pfam databases (E-value ≤ 1 × 10^−5^). InterProScan v5.36 [60] annotated protein domains against InterPro and Pfam, integrating GO terms from InterPro.

For non-coding RNA prediction, miRNAs, snRNAs, and snoRNAs were annotated using the Rfam v14.1 library [61]. tRNAs were identified using tRNAscan-SE v1.3.1 [62], and rRNAs were predicted using Barrnap (0.9) [63].

### 4.9. Whole-Genome Synteny Analysis

MUMmer software (v4.0.0beta) [64] was employed to align the genome sequences of 124Pan, CN14, Lovell, and *P. ferganensis* to the genome sequence of QZM. Then, we extracted the unique fragments of QZM that differed compared with each of the other four genomes.

### 4.10. Genome Evolution, Divergence Time, and Duplication Events

The genomes of Lovell (v2.0), *P. ferganensis*, Chinese cling, 124Pan, CN14, *P. dulcis*, *P. armeniaca*, *P. avium*, and *M. domestica* were downloaded from GDR (https://www.rosaceae.org/ (accessed on 7 July 2023)). The genomes of *O. sativa* and *Arabidopsis thaliana* were downloaded from the National Center for Biotechnology Information (NCBI) (https://www.ncbi.nlm.nih.gov/ (accessed on 3 July 2023)) and The Arabidopsis Information Resource (TAIR) (https://www.arabidopsis.org/ (accessed on 5 July 2023)). The gene sequences in each single-copy gene family were aligned using MAFFT software (v7.511) [65] for multiple sequence alignment. The phylogenetic tree of species was constructed using the maximum likelihood method (ML TREE) in RAxML software (v8.2.13) [66]. The Ks of each homologous gene pair was calculated based on the aforementioned alignment results using the KaKs Calculator software (v3.0) [67]. The divergence time was estimated in MCMCTree software (v4.9; parameters: clock = 3 and model = 0) [68]. The expanded and contracted genes were analyzed using Cafe5 (v5.0.0) [69].

### 4.11. GWASs

A total of 145 peach accessions comprising 29 landrace peaches from Shandong Province and 116 additional peach varieties were employed to perform GWASs for fruit hairs, fruit shape, fruit ripening, and fruit weight. Two-year data for fruit weight in each accession were averaged and then log_2_-transformed to serve as the final trait data in the association panel.

The raw SNP dataset was subjected to a multi-step filtering process to ensure that the data was of high quality. First, hard filtering was applied using the Genome Analysis Toolkit [70] based on standard best practices. Variants were excluded if they met any of the following criteria: quality by depth < 2.0, mapping quality < 40.0, Fisher strand bias > 60.0, strand odds ratio > 3.0, mapping quality rank sum test (MQRankSum) < −12.5, or read position rank sum test (ReadPosRankSum) < −8.0. Subsequently, loci with a missing genotype rate exceeding 20% across individuals were removed. To retain only biallelic SNPs, multiallelic sites were discarded. Rare variants were filtered out by excluding SNPs with a minor allele frequency less than 0.05. Finally, 1,412,216 high-quality SNPs with minor allele frequency > 0.05 were utilized as the genotypic panel for GWAS analysis. The Benjamini–Hochberg method was applied to adjust *p* values for multiple testing [71]. To control for the confounding effects of population structure in GWAS, principal component analysis was performed using the high-quality SNPs. The top principal components 1, 2, 3, and 4 reflecting the genetic structure of the population were incorporated as covariates in the GWAS model to reduce spurious associations caused by population stratification and improve the accuracy and reliability of the detected loci (Figure S3). Principal component analysis was performed using EIGENSOFT to plot the first four principal components [72]. GWAS analysis was then performed for the four traits in the resequencing population using the mixed linear model (MLM) in EMMAX software (v1.0 beta) [73]. Admixture software (v1.32) [74] was utilized for population structure analysis. The significance screening threshold for the four traits in this association analysis was set to 6 [75].

## 5. Conclusions

This study presents a high-quality chromosome-level genome assembly of the QZM landrace peach, an important genetic resource with over 400 years of cultivation history. Our comprehensive genomic analyses reveal several key findings that illuminate the genetic basis of QZM’s distinctive agronomic traits. First, comparative genomics identified 1939 unique genomic fragments containing 418 genes, many of which are associated with disease resistance or stress tolerance pathways. Second, evolutionary analysis demonstrated significant contraction in gene families related to ethylene biosynthesis (*ACOs* and *ETRs*) and fruit softening (*PGs*), providing molecular explanations for QZM’s extremely late-ripening phenotype and improved storage tolerance. Third, GWAS analysis uncovered a novel peak signal on chromosome 2 associated with fruit weight. These findings not only advance our understanding of peach domestication and genome evolution, but they also provide actionable genetic targets for breeding programs seeking to improve fruit quality, extend shelf life, and enhance stress resistance. The QZM genome assembly established in this study serves as a critical foundation for future functional genomics research and the development of superior peach cultivars.

## Figures and Tables

**Table 1:** Length distribution of specific fragments from the QZM ^a^ genome vs. four different peach genomes ^b^.

	Type	Number	Length (bp)
All	<100 bp	409	12,748
	100 bp–1 kb	867	316,920
	1 kb–10 kb	390	1,379,294
	>10 kb	273	7,538,701
Chromosomal Regions	<100 bp	404	12,614
	100 bp–1 kb	847	306,312
	1 kb–10 kb	273	800,539
	>10 kb	47	1,230,589
Non-chromosomal Region	<100 bp	5	134
	100 bp–1 kb	20	10,608
	1 kb–10 kb	117	578,755
	>10 kb	226	6,308,112

^a^ QZM, landrace peach, Qing Zhou Mi. ^b^ Four different peach genomes: 124Pan, CN14, Lovell, and *Prunus ferganensis*.

**Table 2:** Statistics of three gene families across the genomes of three accessions.

Family	QZM ^a^	124Pan ^b^	*Prunus ferganensis*
*ACS*	8	8	8
*ACO*	29	33	31
*ETR*	3	4	4

^a^ QZM, landrace peach, Qing Zhou Mi. ^b^ 124Pan, cultivar peach 124 Pan.

## Data Availability

The raw sequencing data of the QZM genome have been uploaded to the NCBI (https://www.ncbi.nlm.nih.gov/ (accessed on 10 November 2023)) under the data number SAMN37778061. The GWAS resequencing data have been uploaded to the NCBI under the data number SAMN37823607.

## Supplementary Materials

The following supporting information can be downloaded at: https://www.mdpi.com/article/10.3390/plants15071113/s1, Figure S1. Kyoto Encyclopedia of Genes and Genomes (KEGG) terms for genes from the contracted gene families in the ‘Qing Zhou Mi’ (QZM) landrace peach genome. Figure S2. Kyoto Encyclopedia of Genes and Genomes (KEGG) terms for genes from the expanded gene families in the ‘Qing Zhou Mi’ (QZM) landrace peach genome. Figure S3. Principal component analysis plots of the first four principal components (PC1, PC2, PC3, and PC4) for 145 samples. Table S1. Statistical results of repetitive sequences. Table S2. Statistical results of repetitive sequence classification. Table S3. List of 46 positively selected genes. Table S4. List of PG genes across three genomes of peach accessions. Table S5. Information on Qizhoumi and four sequenced accessions (124Pan, CN14, Lovell, and *Prunus ferganensis*). Table S6. Information on the GWAS population.

## Abbreviations

ACO, ACC Oxidase; ACS, ACC Synthase; CN14, Zhongyoutao 14; ETR, Ethylene Receptor; GWAS, Genome-Wide Association Study; FCHL, Feichenghongli; GO, Gene Ontology; GWAS, Genome Wide Association Studies; KEGG, Kyoto Encyclopedia of Genes and Genomes; LHSM, Longhuashuimi; MLM, Mixed Linear Model; PG, Polygalacturonase; QZM, Qing Zhou Mi; RYP1, Rui You Pan 1; SAM, S-adenosylmethionine.
