# Supplementary material for: De Novo Chromosome-Level Genome Assembly of ‘Qing Zhou Mi’ Landrace Peach and Analysis of Late Maturity and Fruit Weight Traits in Peach
Source: Plants (Basel). 2026 Apr 3;15(7):1113. doi: 10.3390/plants15071113 (PMC13074578; doi:10.3390/plants15071113)
Supplement: Supplementary file 1 [file plants-15-01113-s001.zip › Supplementary Figures S1-S3.pdf]

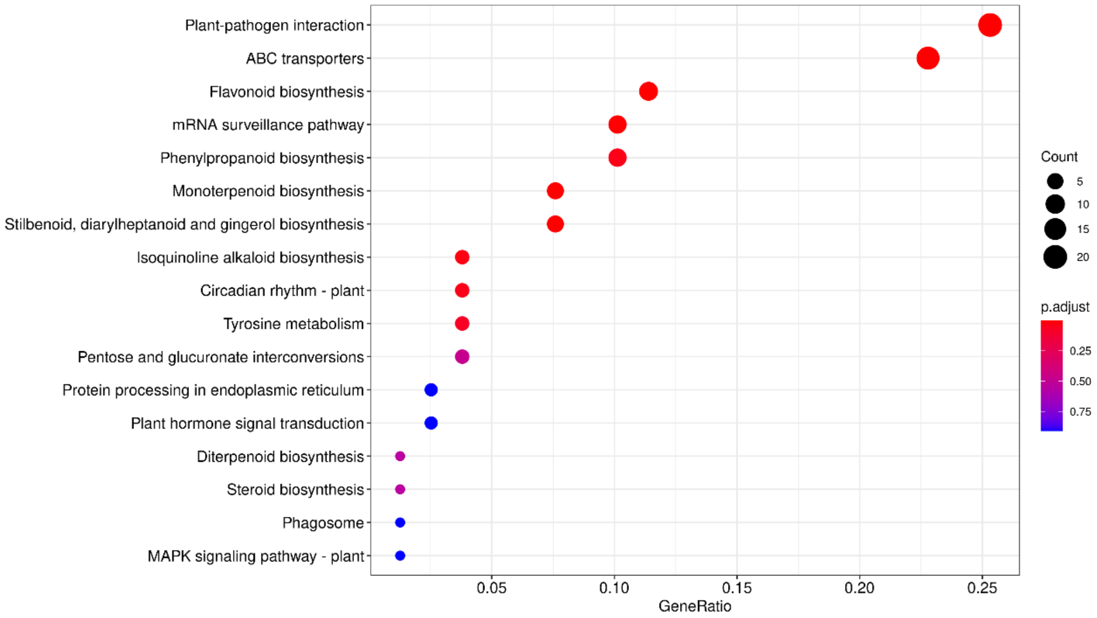

Supplementary Figure S1. Kyoto Encyclopedia of Genes and Genomes (KEGG) terms for genes from the contracted gene families in the 'Qing Zhou Mi' (QZM) landrace peach genome.

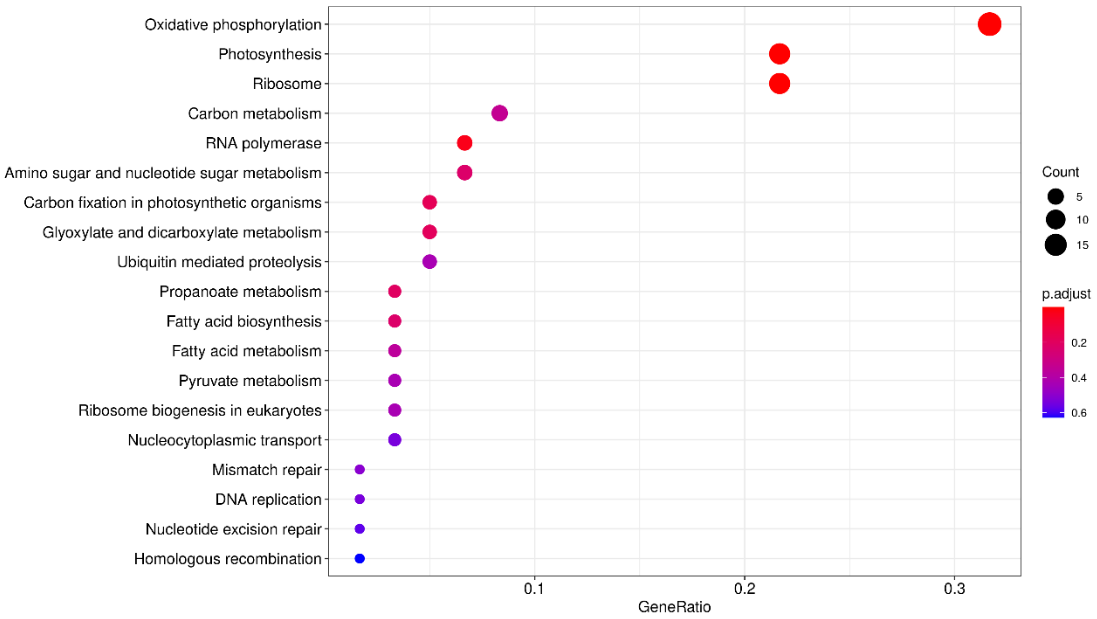

Supplementary Figure S2. Kyoto Encyclopedia of Genes and Genomes (KEGG) terms for genes from the expanded gene families in the 'Qing Zhou Mi' (QZM) landrace peach genome.

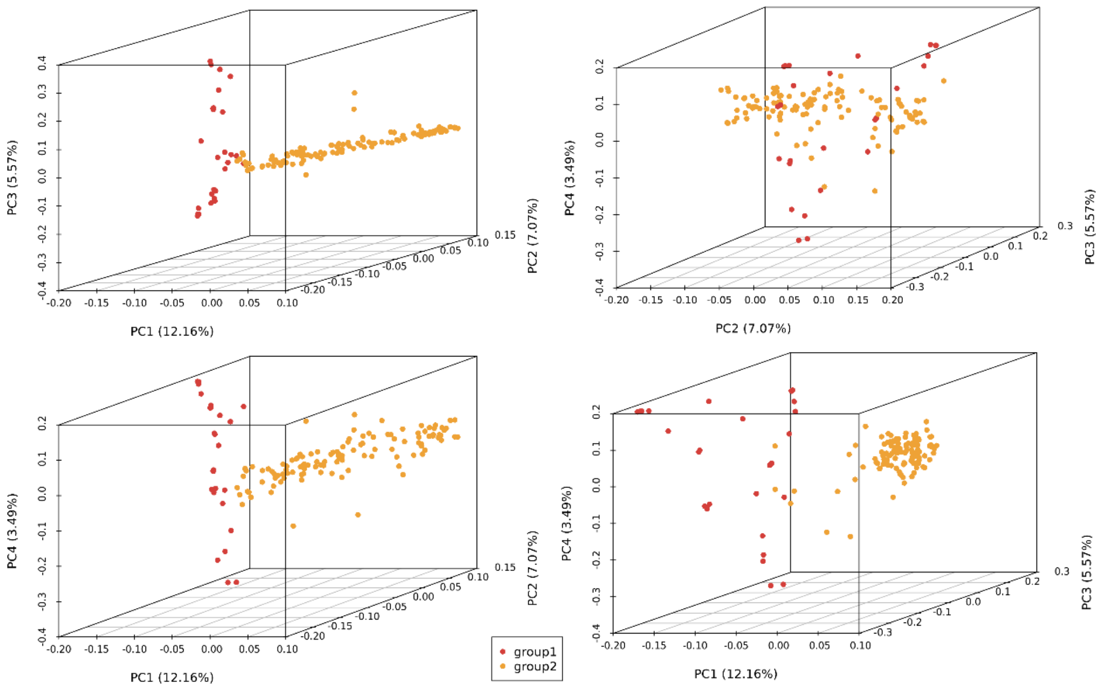

Supplementary Figure S3. Principal Component Analysis plots of the first four principal components (PC1, PC2, PC3, and PC4) for 145 samples. Percentages on axes denote the proportion of genetic variance explained. Group I, 29 landrace peaches originated from 'Qing Zhou Mi' (QZM); Group II, the remaining 116 peach accessions.
