# Supplementary material for: De Novo Chromosome-Level Genome Assembly of ‘Qing Zhou Mi’ Landrace Peach and Analysis of Late Maturity and Fruit Weight Traits in Peach
Source: Plants (Basel). 2026 Apr 3;15(7):1113. doi: 10.3390/plants15071113 (PMC13074578; doi:10.3390/plants15071113)
Supplement: Supplementary file 1 [file plants-15-01113-s001.zip › Supplementary Tables S1-S2.pdf]

Supplementary Table S1. Statistical results of repetitive sequences.

Type	Repeat Size (bp)	Genome (%)
Trf	23,700,801	9.39
Repeatmasker	43,776,511	17.35
Proteinmask	20,275,023	8.03
De novo	66,168,243	26.22
Total	99,164,565	39.29

Supplementary Table S2. Statistical results of repetitive sequence classification.

	RepBase TEs		TE Proteins		De novo		Combined TEs	
	**Length (bp)**	**Genome (%)**	**Length (bp)**	**Genome (%)**	**Length (bp)**	**Genome (%)**	**Length (bp)**	**Genome (%)**
DNA	14,764,853	5.85	5,272,678	2.09	16,970,735	6.72	26,398,589	10.46
LINE	1,651,051	0.65	1,107,373	0.44	2,208,676	0.88	3,547,213	1.41
SINE	28,396	0.01	0	0.00	136,252	0.05	164,276	0.07
LTR	27,576,750	10.93	13,889,788	5.50	34,866,102	13.81	47,369,398	18.77
Satellite	399,895	0.16	0	0.00	427,499	0.17	825,583	0.33
Simple_repeat	0	0.00	0	0.00	0	0.00	0	0.00
Other	580	0.00	0	0.00	0	0.00	580	0.00
Unknown	155,361	0.06	11,412	0.00	12,313,892	4.88	12,477,084	4.94
Total	43,776,511	17.35	20,275,023	8.03	66,168,243	26.22	88,294,489	34.98
